# Influencing factors associated with maternal delivery at home in urban areas: a cross-sectional analysis of the Bangladesh Demographic and Health Survey 2017–2018 data

**DOI:** 10.1186/s41043-023-00428-9

**Published:** 2023-08-21

**Authors:** Khandaker Tanveer Ahmed, Md. Karimuzzaman, Shohel Mahmud, Labiba Rahman, Md. Moyazzem Hossain, Azizur Rahman

**Affiliations:** 1 Department of Statistics, Jahangirnagar University, Savar, Dhaka, 1342 Bangladesh; 2 DREXEL Dornsife School of Public Health, DREXEL University, Philadelphia, PA USA; 3 Department of Statistics, Noakhali Science and Technology University, Noakhali, 3814 Bangladesh; 4 School of Mathematics, Statistics, and Physics, Newcastle University, Newcastle upon Tyne, Tyne and Wear UK; 5 School of Computing, Mathematics and Engineering, Charles Sturt University, Wagga Wagga, NSW 2678 Australia

**Keywords:** Determinants, Delivery at home, Reproductive health, Bangladesh

## Abstract

**Background:**

The associated factors and patterns of giving birth in home settings of rural areas have been extensively studied in Bangladeshi literature. However, urban areas still need to be explored, particularly with recent data. Therefore, the authors aimed to investigate the influential determinants of delivery at home in urban areas of Bangladesh.

**Materials and methods:**

In this study, 1699 urban-dwelling women who had given birth within the previous 60 months of the survey and lived in urban areas were used. The secondary data were extracted from the latest Bangladesh Demographic and Health Survey 2017–2018. Descriptive statistics and logistic regression were applied along with the association among selected variables were examined by the Chi-square test.

**Results:**

Findings depict that 36.49% of women who lived in urban areas of Bangladesh delivered at home, whereas, 63.51% delivered at different govt. and private health care facilities. Women who lived in Chittagong [adjusted odds ratio (AOR) = 2.11, 95% CI 1.24–3.60], Barisal [AOR = 2.05, 95% CI 1.16–3.64] and Sylhet [AOR = 1.92, 95% CI 1.08–3.43] divisions have more likelihood to deliver at home (36.85%). Urban women following Christian religion [AOR = 10.71, 95% CI 1.32–86.68] have higher odds of delivering child at home (0.47%). Urban women having three or more children before her latest delivery (22.37%) and who are employed (29.37%) have more likelihood to deliver at home. However, women aged between 25 and 34 years (43.50%), who have higher education (25.90%), play the role of household head (9.06%), have parity of more than two births (2.24%), and read daily newspapers (68.69%) had a lower chance of delivery at home. Furthermore, women from wealthier families (89.12%) and more antenatal care (ANC) visits (94.93%) were less likely to have a delivery at home.

**Conclusion:**

Despite significant progress in women and reproductive health in Bangladesh, the proportion of delivery in the home in urban areas is alarming and should be emphasized more. The authors believe the identified factors will help design interventions and policy development on this issue.

## Introduction

Child and maternal mortality are one of the most prominent global health problems, particularly in developing countries [1]. In order to combat this situation, the United Nations established the Millennium Development Goals (MDGs) in 2000, with Goal 4 aiming to reduce the under-five mortality rate by two-thirds between 1990 and 2015 [2] and Goal 5 aiming to reduce maternal mortality by 75 percent by the year 2015 and achieve universal access to reproductive health [3, 4]. Every year, an estimated 287,000 women die globally due to pregnancy-related causes, with almost every fatality (99%) occurring in developing nations [5]. Sub-Saharan African and Asian countries experience high maternal mortality ratio (MMR), accounting for 87% of global maternal mortality in 2020 [6]. Maternal mortality in Bangladesh was 1.43 per thousand births in 2015, which was below the Millennium Development Goal. Following that, in 2015, the United Nations developed the Sustainable Development Goals (SDGs), which include improvements in women's health, and a decrease in child and maternal mortality, among other objectives [7]. Concerning maternal mortality, target 3.1 of the Sustainable Development Goals aims to reduce the global MMR to less than 70 per 100,000 live births by the year 2030, and target 3.2 aims to reduce neonatal mortality to at least 12 per 1000 live births and under-5 mortality to at least 25 per 1000 live births by the year 2030 [8–10]. Despite similarities in community characteristics, cultural context, health systems, and geographical proximity, the MMR in the Southeast Asian region has not achieved the SDG's target and shows significant variation across countries [11]. Even though the globe continues to suffer from maternal mortality, with 211 deaths per 100,000 live births in 2017, the MMR in Bangladesh was 173 deaths per 100,000 live births in 2017 [5]. Indonesia ranked third highest in Southeast Asia for maternal mortality in 2017, with a rate of 177 deaths per 100,000 live births, with Myanmar and Laos having higher rate [12]. Over the past few years, there has been a substantial increase in MMR in developing countries, whereas developed countries have experienced only a slight rise [13].

Despite increased worldwide attention in recent years on the essential need to address unmet health needs of pregnant women and children, significant progress in reducing maternal mortality has been noticed as sluggish [14]. Bangladesh, Nepal, India, and Honduras show significant progress in reducing maternal mortality rates through effective health plans and government commitment [15]. Every day, 800 women die from avoidable causes linked to pregnancy and delivery, according to the World Health Organization, with emerging nations accounting for 99 percent of all maternal fatalities [5]. Women in developing countries have a lifetime risk of dying from maternal-related causes about 33 times higher than the risk of dying from maternal-related causes in affluent countries [16]. World Health Organization (WHO) advises that all births be carried out by a skilled birth attendant (SBA), a health professional who is competent in diagnosing and managing problems and providing basic primary care and referral [17, 18]. Bangladesh has made substantial progress in reducing maternal and child mortality, but the SBA-assisted delivery process falls short of expectations [19]. The “health-center intrapartum care approach” for managing labor difficulties and decreasing maternal and newborn mortality is regarded as a key method in which trained professionals handle childbirth and associated complications [20, 21]. Studies in Bangladesh and similar settings emphasize the significant influence of individual and health system factors on maternal death and health facility delivery utilization [22].

Numerous efforts have been made to reduce maternal and neonatal deaths globally in low- and middle-income countries (LMICs) through increased utilization of maternal healthcare facilities [23, 24]. A significant proportion of mothers continue to deliver at home in developing countries, unattended by SBA [21, 25–27]. Additionally, many childbearing women in LMICs, particularly those in South Asia (SA), continue to face barriers to accessing and utilizing maternal healthcare services, such as pregnancy and delivery. As a result, they prefer to deliver at home with the assistance of a traditional birth attendant (TBA) over an SBA. The evidence suggests that the trend toward home births among women in South Africa carries significant risks to the mother’s health and newborn during the postpartum period [28–30]. Several notable risks include mothers abandoning colostrum and breastfeeding practices, mothers neglecting immunization and nutrition supplementation for mother and child, and mothers and children not receiving postnatal care [31–33]. In South Asia, the rate has been reported to be as high as 26 per 1000 births [34]. Optimistically, Bangladesh has made significant progress in the last decade regarding maternal and child mortality reduction. However, the SBA-assisted delivery process falls short of expectations [19]. As of 2017, Bangladesh’s neonatal mortality rate remained as high as 30 per 1000 [35]. A defined consequence was that 64% of rural women planned to give birth at home, and 62% did [36]. Most Bangladeshi studies on this subject either concentrate on rural areas, specific regions, antenatal and postnatal care, skill birth, or C-sections [8, 19, 36–40].

Several factors influencing maternal child delivery at home have been highlighted in previous studies. Women's age has been found to be influential in some related studies [9, 23–26], indicating that different age groups may exhibit varying preferences for home births. Additionally, the resident area or place where women live has emerged as a factor influencing this practice [22], with urban and rural settings potentially leading to different birthing choices. Religion has also been identified as a factor influencing the location of deliveries, with some studies indicating a preference for health facilities among certain religious groups [27]. Women's birth parity has shown to be significant in multiple previous research [27, 33, 38, 41], suggesting that the number of previous births can impact the decision to opt for a home delivery. The wealth index of the respondent's family has been a crucial determining factor in delivery location [17, 22, 26, 42, 43], with economic factors playing a substantial role in the decision-making process. Studies have demonstrated a strong link between the number of antenatal care (ANC) visits and the place of delivery [27, 44, 45], indicating that regular prenatal healthcare may influence the choice of birthing location. Women's education has also been shown to be influential in some related studies [46–49], suggesting that higher education levels may correlate with an increased likelihood of choosing hospital deliveries. Moreover, media exposure, particularly newspaper exposure, has emerged as an influential factor in women's healthcare facility decisions [28, 44, 48, 50–52], indicating that media plays a role in shaping birthing preferences. Finally, women's previous pregnancy experience has been identified as an influential factor in some related studies [27, 38, 41], implying that past birthing experiences can influence the decision to have a home delivery for subsequent pregnancies. Understanding these influential factors can aid in developing targeted interventions and policies to improve maternal and child health outcomes related to home deliveries.

To the best of our knowledge, only a limited number of studies have extensively examined the influential factors associated with home delivery in urban areas, utilizing the most recent data from the Bangladesh Demographic and Health Survey (BDHS). Conducting a study exclusively for urban women in maternal child delivery provides insights into urban–rural disparities, healthcare utilization patterns, population density challenges, socio-economic factors, cultural influences, and the impact of available services. It also helps address the effects of urbanization, design targeted interventions for public health, and explore how migration influences birthing preferences and access to healthcare services. Urban women generally have access to better healthcare facilities, education, and knowledge. Of particular interest is the fact that despite these advantages, some urban women choose home deliveries. This unconventional decision has prompted an investigation into the underlying factors that drive it. Through the exploration of these factors, insights are gained into the distinctive influences that shape maternal delivery choices in urban areas. The findings have the potential to inform targeted interventions aimed at addressing this atypical trend, thereby contributing to improved maternal health outcomes. Understanding these factors can improve maternal and child health outcomes in urban areas. In this article, the authors aimed to explore the factors associated with birthplace, either home or healthcare facility, and reveal the reasons for preferring home birth by a TBA rather than a facility with the help of an SBA in the urban area of Bangladesh.

## Materials and methods

### Study data

The study used secondary data from the 2017–18 BDHS, the eighth of its kind in the country, which began in 1993 and is Bangladesh’s longest-running healthcare survey series. The analysis dataset included only women who lived in urban areas and had a child under 60 months with a defined place of birth of ever-married women aged 15–49 years. The BDHS survey incorporated data from women who gave birth within the 5 years before the survey to obtain recent insights into maternal and child health outcomes and mitigate recall bias. By exclusively considering married women with children under 60 months, the author aimed to emphasize recent maternal and child health outcomes and diminish the potential impact of recall bias. This carefully chosen inclusion criterion ensures that the collected data remains pertinent and current, thereby informing policies and interventions in the domain of maternal and child health. The 2017-18 BDHS data remains relevant due to its status as the latest available dataset for demographic and health information in Bangladesh. Although time has passed, the gradual evolution of socio-economic and cultural factors may suggest the continued applicability of the data to understand maternal delivery practices. Furthermore, its use enables the examination of longer-term trends, contributing to a comprehensive understanding of factors influencing home deliveries in urban areas.

### Sampling design

The BDHS sample for 2017–18 is nationally representative and includes all non-institutional housing units. In 2017–18, the BDHS collected data on five distinct questionnaires, from where only the Woman’s Questionnaire (ever-married women aged 15–49 years) was considered. The first three surveys were adapted from model questionnaires developed for the international DHS-7 Program, taking into account the content of the measures and tailoring them to Bangladesh’s needs and challenges from previous DHS surveys. As a sampling frame, the survey used the Bangladesh Bureau of Statistics (BBS) list of enumeration areas (EAs) from the People’s Republic of Bangladesh’s 2011 Population and Housing Census. The principal sampling unit (PSU) for the survey is the associate EA, which contains 120 households. The survey sampled households in two stages. A total of 675 Enumeration Areas (EAs) were selected for this study, comprising 250 EAs in urban areas and 425 EAs in rural areas. The selection was carried out using a likelihood proportional to EA size, ensuring a representative sample from both urban and rural regions. BBS collected the sample during the first stage, adhering to the DHS team’s guidelines. All selected EAs conducted a comprehensive home listing operation to establish a sampling frame for the second stage of household selection. A scientific sample of thirty families per EA was chosen in the second round of sampling to generate statistically reliable estimates of key demographic and health variables for the entire country, individual urban and rural areas, and each of the eight divisions. As a result of this arrangement, a total of 20,250 residential households were chosen. Around 20,100 never-married women between the ages of 15 and 49 were expected to complete the interviews.

Furthermore, a survey was conducted on a subset of households, around 7–8 homes per designated area (EA), to measure the weight and height of two specific groups of women: those who had ever been married and were over the age of fifty, and those who had never been married and were over the age of eighteen. In addition, all women aged eighteen and above from these households underwent testing for blood pressure and glucose levels, within the same family units. To maintain the credibility of the data and minimize biases stemming from incomplete information, the study purposefully removed any missing data related to the study variables. By concentrating solely on complete cases, which included records of women who had given birth within the last 60 months, the researchers ensured a robust analysis, resulting in dependable and definitive conclusions. The study's findings were based on a total of 1699 records that met these specific criteria. The DHS program granted permission for the study to use the BDHS 2017–18 data.

### Study variables

This study’s outcome variable was home delivery. Within the BDHS woman’s questionnaire, this variable was labeled as “place of delivery”. The responses to this question were classified as 0 for health facility delivery and 1 for home delivery. Deliveries that took place in respondent’s homes and different homes were marked as home delivery. On the contrary, births at government health centers/clinics, medical hospitals, community clinics, specialized hospitals, public, private, and non-governmental organization (NGO) hospitals, maternity homes, and other health facilities were classified as health facility deliveries. A total of fifteen explanatory variables were considered for this study with keeping insights from similar studies [8, 16, 17, 29, 53, 54]. Table 1 summarizes the variables.

**Table 1:** Explanatory variables

Variable	Description	Category
Age	Age in 10-year groups	15–24, 25–34,35–44, 45 or more
Division	Division	Barisal, Chittagong, Dhaka, Khulna, Mymensingh, Rangpur, Rajshahi, Sylhet
Religion	Religion	Islam, Hinduism, Buddhism, Christianity
Education	Respondent’s highest education level	Not educated, Primary, Secondary, Higher
Husband’s education	Husband/partner’s education level	Not educated, Primary, Secondary, Higher
Sex of household head	Sex of household head	Male, Female
Health related decisions	Person who usually decides on respondent’s health care	Respondent alone, respondent with others, others at home
Respondent’s employment	Respondent currently working	Employed, Unemployed
Parity	Parity at sterilization	Two or less, three or more
Wealth index	Wealth index combined	Poorest, Poorer, Middle, Richer, Richest
ANC visits	Number of antenatal visits during pregnancy	No visits, One visit, Two visits, Three visits, Four visits, Five or more
Distance to health facilities	Getting medical help for self: distance to health facility	Not a big problem, Big problem
Read newspaper	Frequency of reading newspaper or magazine	Less than once a week, At least once a week, Almost everyday
Watch TV	Frequency of watching television	Less than once a week, At least once a week, Almost everyday
No. of children	Number of living children	0, 1, 2, 3, 4, 5, 6 or more

### Data processing and statistical analyses

All variables in the study were subjected to frequency analysis. Bivariate analysis was used to demonstrate how the frequency of home delivery is proportional to the respondents’ socio-demographic characteristics at a 95% confidence level. A multicollinearity test was performed on all statistically significant (p ≤ 0.05) variables using the variance inflation factor (VIF). Conducting a multicollinearity test is crucial in the study to ensure accurate and distinct evaluation of the individual effects of predictor variables on maternal home delivery in urban areas, enhancing the reliability and validity of the results. No collinearity was detected between the explanatory variables with a mean VIF of 1.38 (maximum VIF = 1.99, minimum VIF = 1.03). Then multivariable analysis was used, employed as binary logistic regression. Only variables with a declared statistical significance of P ≤ 0.05 were included in the bivariate regression analysis. Adjusted odds ratios at 95% confidence intervals demonstrate their level of precision. To analyze the data, Stata version 16.0 was used.

## Results

A total of 1699 records of urban women were included in this study who were compatible with conducting this study by having all the cases of the required variables. It was found that 36.49 percent of the urban women had home delivery for their children in the last 60 months. Most of the women aged between 15 to 24 years (49.38%) and 25 to 34 years (43.5%) were from Dhaka (24.31%), the capital of Bangladesh. 91.76% of the women were Muslim and had studied for at least secondary level (44.08%). Table 2 summarizes the socio-demographic data and the variables associated with the delivery location.

**Table 2:** Frequency analysis of respondent’s socio-demographic information and factors related to child delivery place

Variables	Frequency	Percentage
*Delivery place*		
Health facility	1079	63.51
Home	620	36.49
*Age (Years)*		
15–24	839	49.38
25–34	739	43.50
35–44	117	6.89
45 or more	4	0.24
*Division*		
Rangpur	163	9.59
Dhaka	413	24.31
Barisal	160	9.42
Khulna	188	11.07
Mymensingh	154	9.06
Rajshahi	155	9.12
Chittagong	265	15.6
Sylhet	201	11.83
*Religion*		
Islam	1559	91.76
Hinduism	124	7.3
Buddhism	8	0.47
Christianity	8	0.47
*Education*		
Not educated	98	5.77
Primary	412	24.25
Secondary	749	44.08
Higher	440	25.9
*Husband’s education*		
Not educated	170	10.01
Primary	494	29.08
Secondary	559	32.9
Higher	476	28.02
*Sex of household head*		
Male	1545	90.94
Female	154	9.06
*Health related decisions*		
Respondent alone	144	8.48
Respondent with others	1159	68.22
Others at home	396	23.31
*Respondent’s employment*		
Unemployed	1200	70.63
Employed	499	29.37
*Parity*		
≤ 2	1661	97.76
3 or more	38	2.24
*Wealth index*		
Poorest	185	10.89
Poorer	140	8.24
Middle	231	13.6
Richer	442	26.02
Richest	701	41.26
*ANC visits*		
No visits	86	5.06
One visit	154	9.06
Two visits	217	12.77
Three visits	235	13.83
Four visits	210	12.36
Five or more visits	797	46.91
*Distance to health facilities*		
Big problem	557	32.78
Not a big problem	1142	67.22
*Read newspaper*		
Less than once a week	1396	82.17
At least once a week	183	10.77
Almost everyday	120	7.06
*Watch TV*		
Less than once a week	415	24.43
At least once a week	117	6.89
Almost everyday	1167	68.69
*No. of children*		
0	19	1.12
1	719	42.32
2	581	34.2
3	264	15.54
4	79	4.65
5	21	1.24
6 or more	16	0.94

Table 3 summarizes the bivariate analysis conducted between the child’s delivery location and the socio-demographic characteristics of the study population, and some factors associated with delivery location selection. Age, division, religion, education, husband’s education, respondent’s employment, household head’s sex, parity, wealth index, ANC visits, distance to the health facility, reading newspaper, watching TV, and number of children were all found to be associated with home delivery of urban women. However, health-related decisions had no significant association with the delivery location.

**Table 3:** Association between place of delivery and variables related to the child delivery place

Variables	Delivery in health facility		Home delivery		Chi-square (*p*-value)
	Frequency	Percentage	Frequency	Percentage	
*Age (Years)*					
15–24	503	60.0	336	40.0	9.799* (0.020)
25–34	498	67.4	241	32.6	
35–44	76	65.0	41	35.0	
45 or more	2	50.0	2	50.0	
*Division*					
Rangpur	116	71.2	47	28.8	47.265*** (< 0.001)
Dhaka	286	69.2	127	30.8	
Barisal	85	53.1	75	46.9	
Khulna	136	72.3	52	27.7	
Mymensingh	95	61.7	59	38.3	
Rajshahi	110	71.0	45	29.0	
Chittagong	138	52.1	127	47.9	
Sylhet	113	56.2	88	43.8	
*Religion*					
Islam	982	63.0	577	37.0	22.374** (0.001)
Hinduism	94	75.8	30	24.2	
Buddhism	1	12.5	7	87.5	
Christianity	2	25.0	6	75.0	
*Education*					
Not educated	33	33.7	65	66.3	258.560*** (< 0.001)
Primary	167	40.5	245	59.5	
Secondary	486	64.9	263	35.1	
Higher	393	89.3	47	10.7	
*Husband’s education*					
Not educated	69	40.6	101	59.4	206.731 (< 0.001***)
Primary	231	46.8	263	53.2	
Secondary	368	65.8	191	34.2	
Higher	411	86.3	65	13.7	
*Sex of household head*					
Male	970	62.8	575	37.2	3.863* (0.049)
Female	109	70.8	45	29.2	
*Health related decisions*					
Respondent alone	91	63.2	53	36.8	3.13 (0.210)
Respondent with others	751	64.8	408	35.2	
Others at home	237	59.8	159	40.2	
*Respondent’s employment*					
Unemployed	807	67.3	393	32.3	24.687*** (< 0.001)
Employed	272	54.5	227	45.5	
*Parity*					
≤ 2	1045	62.9	616	37.1	11.308** (0.001)
3 or more	34	89.5	4	10.5	
*Wealth index*					
Poorest	37	20.0	148	80.0	302.301*** (< 0.001)
Poorer	68	48.6	72	51.4	
Middle	119	51.5	112	48.5	
Richer	269	60.9	173	39.1	
Richest	586	83.6	115	16.4	
*ANC visits*					
0	10	11.6	76	88.4	276.580*** (< 0.001)
1	59	38.3	95	61.7	
2	93	42.9	124	57.1	
3	141	60.0	94	40.0	
4	139	66.2	71	33.8	
5 or more	637	79.9	160	20.1	
*Distance to health facilities*					
Big problem	291	52.2	266	47.8	45.365*** (< 0.001)
Not a big problem	788	69.0	354	31.0	
*Read newspaper*					
Less than once a week	816	58.5	580	41.5	93.314*** (< 0.001)
At least once a week	148	80.9	35	19.1	
Almost everyday	115	95.8	5	4.2	
*Watch TV*					
Less than once a week	166	40.0	249	60.0	141.583*** (< 0.001)
At least once a week	67	57.3	50	42.7	
Almost everyday	846	72.5	321	27.5	
*No. of children*					
0	14	73.7	5	26.3	66.961*** (< 0.001)
1	502	69.8	217	30.2	
2	373	64.2	208	35.8	
3	151	57.2	113	42.8	
4	31	39.2	48	60.8	
5	6	28.6	15	71.4	
6 or more	2	12.5	14	87.5	

*p < 0.05, ** *p* < 0.01, *** *p* < 0.001

Table 4 contains the outcome of the multivariate logistic regression which was performed to check which factors were affecting home delivery among urban women. The model achieved a pseudo R^2^ value of 0.285, indicating its explanatory power. Additionally, the likelihood ratio chi-square test resulted in a value of 634.8 with a p-value less than 0.001, suggesting a statistically significant relationship in the model. Urban women aged 25–34 years [AOR = 0.58, 95% CI 0.42–0.80] were less likely to deliver at home in comparison to women aging between 15 to 24 years. Women living in Chittagong division [AOR = 2.11, 95% CI 1.24–3.60], Barisal division [AOR = 2.05, 95% CI 1.16–3.64] and Sylhet division [AOR = 1.92, 95% CI 1.08–3.43] had higher odds, almost double, of delivering in home than women in Rangpur division. Hindu women had reduced odds of delivering at home (AOR = 0.44, 95% CI 0.26–0.74) compared to Muslim women. In contrast, Christian women were ten times more likely to opt for a home birth (AOR = 10.71, 95% CI 1.32–86.68) than Muslim women. Women who received higher education had less likelihood [AOR = 0.33, 95% CI 0.16–0.66] of home delivery than uneducated women. If the household head was a female, then it was less likely [AOR = 0.57, 95% CI 0.37–0.89] for women to have a home delivery of child. Employed women were more likely [AOR = 1.63, 95% CI 1.23–2.16] to home delivery. With parity of more than 2 birth, women were very less likely [AOR = 0.08, 95% CI 0.03–0.27] to delivering child at home than the women with 2 or less birth parity at sterilization. Poorer families [AOR = 0.42, 95% CI 0.24–0.74], middle wealthy families [AOR = 0.43, 95% CI 0.25–0.72], richer families [AOR = 0.3, 95% CI 0.18–0.50] and the richest families [AOR = 0.19, 95% CI 0.11–0.34] were less likely to have home delivery of child comparing to poorest families. Women who had ANC visits once [AOR = 0.27, 95% CI 0.12–0.62], twice [AOR = 0.29, 95% CI 0.13–0.64], thrice [AOR = 0.18, 95% CI 0.08–0.39], four times [AOR = 0.17, 95% CI 0.07–0.37] and more than four visits [AOR = 0.10, 95% CI 0.04–0.21] had less odds of delivering at home than women with no ANC visits. Women reading newspaper daily had less likelihood [AOR = 0.29, 95% CI 0.11–0.78] of home delivery. If a woman had 3 children before her latest delivery, then the woman was three times more likely [AOR = 3.55, 95% CI 1.03–12.28] to deliver at home. Women with 4 children were five times more likely [AOR = 5.74, 95% CI 1.49–22.03] and with more than 5 children were fifteen times more likely [AOR = 15.15, 95% CI 1.35–169.92] to have their delivery of child at home comparing to the women who had no children prior to their delivery.

**Table 4:** Assessing the factors influencing home delivery among urban women using multivariate logistic regression

Variable	Adjusted odds ratio (AOR)	95% Confidence interval	
		Lower bound	Upper bound
*Age (Years)*			
15–24 (ref.)	1		
25–34	0.58**	0.42	0.80
35–44	0.55	0.28	1.06
45 or more	0.08	0.01	1.33
*Division*			
Rangpur (ref.)	1		
Dhaka	1.49	0.89	2.49
Barisal	2.05*	1.16	3.64
Khulna	1.20	0.68	2.13
Mymensingh	1.52	0.85	2.70
Rajshahi	1.27	0.71	2.28
Chittagong	2.11**	1.24	3.60
Sylhet	1.92*	1.08	3.43
*Religion*			
Islam (ref.)	1		
Hinduism	0.44**	0.26	0.74
Buddhism	9.97	0.48	206.40
Christianity	10.71*	1.32	86.68
*Education*			
Not educated (ref.)	1		
Primary	0.99	0.57	1.74
Secondary	0.64	0.36	1.11
Higher	0.33**	0.16	0.66
*Husband’s education*			
Not educated (ref.)	1		
Primary	0.96	0.62	1.47
Secondary	0.80	0.51	1.24
Higher	0.78	0.44	1.36
*Sex of household head*			
Male (ref.)	1		
Female	0.57*	0.37	0.89
*Respondent’s employment*			
Unemployed (ref.)	1		
Employed	1.63**	1.23	2.16
*Parity*			
≤ 2 (ref.)	1		
3 or more	0.08***	0.03	0.27
*Wealth index*			
Poorest (ref.)	1		
Poorer	0.42**	0.24	0.74
Middle	0.43**	0.25	0.72
Richer	0.30***	0.18	0.50
Richest	0.19***	0.11	0.34
*ANC visits*			
No visits (ref.)	1		
One visit	0.27**	0.12	0.62
Two visits	0.29**	0.13	0.64
Three visits	0.18***	0.08	0.39
Four visits	0.17***	0.07	0.37
Five or more visits	0.10***	0.04	0.21
*Distance to health facilities*			
Big problem (ref.)	1		
Not a big problem	0.97	0.74	1.26
*Read newspaper*			
Less than once a week (ref.)	1		
At least once a week	0.88	0.55	1.40
Almost everyday	0.29*	0.11	0.78
*Watch TV*			
Less than once a week (ref.)	1		
At least once a week	0.95	0.57	1.60
Almost everyday	0.84	0.60	1.16
*No. of children*			
0 (ref.)	1		
1	1.80	0.55	5.91
2	2.64	0.80	8.75
3	3.55*	1.03	12.28
4	5.74*	1.49	22.03
5	4.94	0.89	27.44
6 or more	15.15*	1.35	169.92

*p < 0.05, ** *p* < 0.01, *** *p* < 0.001

## Discussion

Findings of this study revealed that older women had lower odds of giving birth at home than younger women aged 15–24 years, implying that older women prefer to give birth in health care facilities, which is consistent with the findings of related studies [9, 23–26, 55]. Moreover, women in urban regions of Barisal, Chittagong, and Sylhet divisions were more likely to give birth at home than women in the Rangpur division, which is surprising given that Chittagong is Bangladesh’s second-largest city and these three cities are more developed than others. Another study [22] also observed the same thing in Chittagong and Barisal, claiming that healthcare delivery in these cities was poor [8]. Furthermore, the Hindu faith had a smaller proclivity for home delivery than Islam, but Christians had a tenfold increase. Likewise, similar studies found that Muslim women avoid institutional deliveries and have a deficient proportion of their babies delivered in health facilities [27].

Moreover, women who had more than two births at the time of sterilization had a far lower odds of giving birth at home than women who had two or fewer births at sterilization. Because of their greater experience and understanding of health issues, women with higher birth parity are more likely to want to deliver in a health care facility rather than at home, according to multiple previous research [27, 33, 38, 41]. On the other hand, studies [56, 57] claim that more parity leads to lower healthcare delivery. However, the wealth index of the respondent’s family was a crucial determining factor in delivery location, and the study clearly showed that the wealthier the families were, the less likely they were to have home delivery, which is also consistent with other related studies of both Bangladesh and other countries [17, 22, 26, 40, 42, 43, 58]. This finding also comes in line with several studies where it was maintained that wealthier families and women are more likely to deliver in health care institutions than more impoverished families [30, 45, 51, 52, 55].

Interestingly, our study demonstrated a strong link between ANC visits and institutional delivery, similar to several other studies conducted in Bangladesh [27, 44, 45, 55]. More ANC visits influence women’s decision about where to give birth, and ANC-visited women prefer to give birth at a hospital [45, 59, 60]. The likelihood of giving birth at home decreases as the number of ANC visits increases. Sufficient ANC visits inform women about health risks during delivery and the availability of expert delivery personnel, influencing them to choose institutional delivery. They also educate mothers about the advantages of health facility delivery as a critical juncture [46–49]. In contrast, reading the newspaper every day enlightens women on institutional deliveries through important health-related messages released by government and NGOs and assists them in comprehending the benefits and drawbacks of different delivery options [8]. Moreover, women’s healthcare facilities were influenced by media exposure, particularly newspaper exposure [28, 44, 48, 50–52, 61]. However, the number of children before the current delivery was found to be a significant factor that influences home delivery, with a higher number of children before the current delivery showing a higher likelihood of delivering at home, in this study. Likewise, women who have had previous pregnancies are more secure in their ability to deliver, so they may feel safe having a regular delivery at home [27, 38, 41].

However, Islam and Christianity may be more religiously rigid than Hinduism when it comes to childbirth. Disparities in health care delivery may occur due to religious affiliation, as shown in Ghana [17, 53] and Nepal [26]. The intriguing finding of Christian women in urban areas favoring home deliveries requires a multifaceted exploration. Examining religious doctrines and cultural norms could unveil specific influences shaping their choices. Furthermore, assessing healthcare accessibility tailored to diverse religious needs may reveal nuanced factors driving this trend. This comprehensive approach would untangle the complex interaction of religious identity, cultural context, and healthcare dynamics, yielding a more profound understanding of the observed association. The delivery location may have been influenced by a specific and religious solid conviction [29, 30], but it cannot be claimed that this is the actual reason. In contrast, research conducted in Bangladesh [52, 62, 63], Ghana [46], Kenya [30], and Ethiopia found that women with higher levels of education had a lower likelihood of giving birth at home [31, 50, 64]. Women who have had formal education are more aware of maternal risks and healthcare resources, thus they choose institutional deliveries in order to have a safe delivery [23, 58, 61]. Additionally, households headed by a woman were less likely than those headed by a man to deliver a child at home, indicating that women are more apprehensive about health-related issues during labor and prefer to give birth in hospitals. Additionally, employed women were more likely to give birth at home than unemployed women, which may contradict the notion that pregnant women in better financial circumstances are more likely to give birth in hospitals [32–34, 40]. However, the autonomy and freedom to make healthcare decisions may have encouraged employed women to give birth at home [19, 35, 36, 65]. The contrasting findings, where wealthier families exhibit lower home delivery rates while employed women, often financially better off, show higher rates of home deliveries, highlight a complex interplay of factors. This apparent contradiction could stem from nuanced dynamics influenced by cultural norms, time constraints, and regional variations within urban areas. Considering these intricate interactions may help reconcile the disparity and provide a more comprehensive understanding of how wealth, employment, and other contextual elements collectively shape maternal delivery decisions. Moreover, due to experience gained from past deliveries, some women with previous birth records may rely on an untrained traditional birth attendant for their delivery at home [19].

This study has significant implications for improving maternal and child health outcomes in urban areas. The identification of influential factors impacting delivery place choices among urban women, including age, education level, religion, wealth index, and ANC visits, empowers policymakers and healthcare providers to develop targeted interventions for better maternal and child health. The findings align with various Sustainable Development Goals (SDGs) related to health, gender equality, education, and decent work. Strengthening maternal healthcare services, addressing socio-economic disparities, promoting women's education, and fostering informed decision-making during pregnancy are potential avenues through which this research can support the goals of the Bangladesh Government and enhance maternal and child health in urban settings. The preference for home deliveries in urban areas is influenced by cultural and social factors, such as gender norms, community beliefs, and perceptions of childbirth. Gender inequalities may limit women's autonomy in decision-making, while community traditions and the role of traditional birth attendants may perpetuate the preference for home births. Some women may perceive home deliveries as safer and more private, while negative experiences or mistrust in institutional care may discourage hospital choices. Addressing these factors requires community engagement, awareness-raising, culturally sensitive healthcare services, and support for women's reproductive choices to promote safer and healthier childbirth experiences in urban settings.

The findings of the study on determinants of home deliveries in urban areas of Bangladesh have important implications for rural areas and other countries. In the context of rural areas in Bangladesh, the identified determinants can offer insights into shared factors influencing home deliveries due to similar cultural norms, limited healthcare access, and socio-economic challenges [8, 19, 36–38]. Likewise, in other low and middle-income countries (LMICs), the study's findings can be compared with relevant literature to uncover common trends and challenges in promoting institutional deliveries [28–30]. Additionally, the study's focus on urban areas provides an opportunity to draw comparisons with similar settings in other countries with comparable socio-economic backgrounds, thereby enriching the understanding of universal and context-specific factors shaping delivery choices [17, 53]. Notably, the importance of access to maternal healthcare facilities and the impact of socio-economic factors, as revealed in the study, can resonate with other regions facing similar challenges [17, 22, 26, 42, 43]. Furthermore, the study's insights into the influence of cultural and religious factors on delivery choices, exemplified by the higher odds of home deliveries among Christian women, can serve as a basis for developing culturally sensitive strategies to promote institutional deliveries in diverse settings [27]. Conducting a systematic literature review to compare the study findings with relevant literature can strengthen the external validity of the research and enhance its applicability in various contexts.

### Limitations and future scopes

The study has some limitations due to its reliance on secondary data (BDHS 2017–18), potentially affecting data quality and nuanced exploration. Additionally, potential biases, such as selection and information bias, may impact the generalizability of findings. To address these limitations, incorporating qualitative methods like interviews or focus groups would provide valuable context and deeper insights into delivery place choices among urban women. Combining quantitative and qualitative data could lead to more robust conclusions. The urban focus may limit generalization to rural areas and other countries, thus future research should include comparative analyses of delivery place choices in both urban and rural settings in Bangladesh and explore factors influencing delivery choices globally. Triangulating quantitative data with qualitative research will enhance understanding across diverse contexts. Another limitation is the lack of sufficient data in BDHS to address potential confounders, such as healthcare service quality, infrastructure accessibility, and skilled birth attendants. This may limit the study's ability to fully capture their impact on home deliveries in urban areas, warranting cautious interpretation of findings. To overcome the limitation of cross-sectional data, referencing data from multiple time points, particularly through longitudinal studies, is crucial. Longitudinal data allows for better analysis of temporal patterns and impacts of interventions, leading to more informed policymaking and a deeper understanding of developments over time.

Based on the research findings, specific interventions and policy recommendations include improving access to quality healthcare facilities, strengthening maternal health services, addressing socio-economic disparities, increasing awareness and education, involving skilled birth attendants, collaborating with religious and community leaders, implementing conditional cash transfer programs, and conducting regular monitoring and evaluation. Longitudinal research is essential to track intervention effectiveness over time and inform evidence-based decision-making. These efforts aim to promote institutional deliveries and enhance maternal healthcare in urban areas of Bangladesh.

## Conclusion

This study used BDHS 2017–18 data to identify several significant factors that influence urban women’s delivery places, including age, division, religion, women’s education level, employment status, household head’s sex, parity, ANC visits, wealth index, daily newspaper reading, and the previous number of children prior to latest delivery. In conclusion, this study reveals diverse factors influencing delivery location choices among urban women in Bangladesh. Despite the availability of healthcare facilities, a significant number still prefer home deliveries. These factors include regional divisions, religion, family size, employment, age, education, household roles, parity, and access to information. To improve maternal health outcomes, policymakers and healthcare providers must address this complexity and develop targeted strategies. Efforts should focus on promoting antenatal care utilization and empowering women through maternal care education and economic opportunities. By understanding the drivers behind home deliveries and identifying vulnerable groups, interventions can prioritize maternal and infant well-being. Initiatives promoting institutional deliveries, safe birthing environments, and better healthcare accessibility can reduce maternal mortality and enhance maternal health in urban areas. In essence, a multi-dimensional approach involving collaboration between policymakers, healthcare professionals, community leaders, and women themselves is essential to ensure safer and informed delivery choices for urban women in Bangladesh, advancing maternal and child health while fostering healthier communities.

## Data Availability

The data set used in this study will be available from the website The DHS Program. In order to gain access to the data files, you have to complete the registration. The data set is available from the following link http://dhsprogram.com/data/available-datasets.cfm.

## References

[1] You D, Hug L, Ejdemyr S, Idele P, Hogan D, Mathers C (2015). Global, regional, and national levels and trends in under-5 mortality between 1990 and 2015, with scenario-based projections to 2030: a systematic analysis by the UN Inter-agency Group for Child Mortality Estimation. Lancet.

[2] WHO (World Health Organization). WHO|Millennium Development Goals (MDGs). World Heal. Organ; 2016.

[3] Alkema L, Chou D, Hogan D, Zhang S, Moller AB, Gemmill A (2016). Global, regional, and national levels and trends in maternal mortality between 1990 and 2015, with scenario-based projections to 2030: a systematic analysis by the un Maternal Mortality Estimation Inter-Agency Group. Lancet.

[4] Brizuela V, Tunçalp Ö (2017). Global initiatives in maternal and newborn health. Obstet Med.

[5] WHO, UNICEF, UNDFPA, Group WB. Trends in maternal mortality: 2000 to 2017: estimates. WHO, UNICEF, UNFPA, World Bank Gr. United Nations Popul. Div; 2019.

[6] WHO. Maternal mortality [Internet]. 2023. Available from: https://www.who.int/news-room/fact-sheets/detail/maternal-mortality

[7] Kruk ME, Gage AD, Arsenault C, Jordan K, Leslie HH, Roder-DeWan S (2018). High-quality health systems in the Sustainable Development Goals era: time for a revolution. Lancet Global Heal.

[8] Badiuzzaman M, Murshed SM, Rieger M (2020). Improving maternal health care in a Post conflict setting: evidence from Chittagong hill tracts of Bangladesh. J Dev Stud.

[9] United Nations Sustainable Development Goals. UNSDG Development Goals. United Nations Stats; 2019.

[10] World Health Organization. Primary health care on the road to universal health coverage 2019 monitoring report. World Heal. Organ; 2019.

[11] Herwansyah H, Czabanowska K, Kalaitzi S, Schröder-Bäck P (2022). The utilization of maternal health services at primary healthcare setting in Southeast Asian countries: a systematic review of the literature. Sex Reprod Healthc.

[12] Mohamed AA, Bocher T, Magan MA, Omar A, Mutai O, Mohamoud SA (2021). Experiences from the field: a qualitative study exploring barriers to maternal and child health service utilization in IDP settings Somalia. Int J Womens Health.

[13] Damayanti NA, Wulandari RD, Ridlo IA (2023). Maternal health care utilization behavior, local wisdom, and associated factors among women in urban and rural areas, Indonesia. Int J Womens Health.

[14] Dol J, Hughes B, Bonet M, Dorey R, Dorling J, Grant A (2022). Timing of maternal mortality and severe morbidity during the postpartum period: a systematic review. JBI Evid Synth.

[15] Patrick M, Zaman MSU, Afzal G, Mahsud M, Hanifatu MN (2022). Factors that affect maternal mortality in Rwanda: a comparative study with India and Bangladesh. Comput Math Methods Med.

[16] Chernet AG, Dumga KT, Cherie KT (2019). Home delivery practices and associated factors in Ethiopia. J Reprod Infertil.

[17] Khan KS, Wojdyla D, Say L, Gülmezoglu AM, Van Look PF (2006). WHO analysis of causes of maternal death: a systematic review. Lancet.

[18] WHO. Making pregnancy safer: the critical role of the skilled attendant. A joint statement by WHO, ICM and FIGO. Geneva, Switz WHO; 2004.

[19] Sarker BK, Rahman M, Rahman T, Hossain J, Reichenbach L, Mitra DK (2016). Reasons for preference of home delivery with traditional birth attendants (TBAs) in rural Bangladesh: a qualitative exploration. PLoS ONE.

[20] Filippi V, Ronsmans C, Campbell OM, Graham WJ, Mills A, Borghi J (2006). Maternal health in poor countries: the broader context and a call for action. Lancet.

[21] Gabrysch S, Campbell OMR (2009). Still too far to walk: literature review of the determinants of delivery service use. BMC Pregnancy Childbirth.

[22] Huda TM, Chowdhury M, El AS, Dibley MJ (2019). Individual and community level factors associated with health facility delivery: a cross sectional multilevel analysis in Bangladesh. PLoS ONE.

[23] Silver KL, Singer PA (2014). SDGs: start with maternal, newborn, and child health cluster. Lancet.

[24] Victora CG, Requejo JH, Barros AJD, Berman P, Bhutta Z, Boerma T (2016). Countdown to 2015: a decade of tracking progress for maternal, newborn, and child survival. Lancet.

[25] Adegoke AA, Van Den Broek N (2009). Skilled birth attendance-lessons learnt. BJOG Int J Obstet Gynaecol.

[26] Koblinsky M, Matthews Z, Hussein J, Mavalankar D, Mridha MK, Anwar I (2006). Going to scale with professional skilled care. Lancet.

[27] Montagu D, Yamey G, Visconti A, Harding A, Yoong J (2011). Where do poor women in developing countries give birth? A multi-country analysis of demographic and health survey data. PLoS ONE.

[28] Ahinkorah BO (2020). Non-utilization of health facility delivery and its correlates among childbearing women: a cross-sectional analysis of the 2018 Guinea demographic and health survey data. BMC Health Serv Res.

[29] Kifle MM, Kesete HF, Gaim HT, Angosom GS, Araya MB (2018). Health facility or home delivery? Factors influencing the choice of delivery place among mothers living in rural communities of Eritrea. J Health Popul Nutr.

[30] Wanjira C, Mwangi M, Mathenge E, Mbugua G, Ng’ang’a Z (2011). Delivery practices and associated factors among mothers seeking child welfare services in selected health facilities in Nyandarua South District, Kenya. BMC Public Health.

[31] Darega B, Dida N, Tafese F, Ololo S (2016). Institutional delivery and postnatal care services utilizations in Abuna Gindeberet district, West Shewa, Oromiya Region, Central Ethiopia: a community-based cross sectional study. BMC Pregnancy Childbirth.

[32] Jafree SR, Zakar R, Mustafa M, Fischer F (2018). Mothers employed in paid work and their predictors for home delivery in Pakistan. BMC Pregnancy Childbirth.

[33] Kaul S, You W, Boyl KJ. Delivery at Home Versus Delivery at a Health Care Facility – A Case Study of Bihar. Sel Pap Prep Present Agric Appl Econ Assoc 2012 AAEA Annu Meet Seattle, Washington, 2012;2012:1–22.

[34] Poudel S, Ghimire PR, Upadhaya N, Rawal L (2020). Factors associated with stillbirth in selected countries of South Asia: a systematic review of observational studies. PLoS ONE.

[35] Rahman AE, Hossain AT, Siddique AB, Jabeen S, Chisti MJ, Dockrell DH (2021). Child mortality in Bangladesh—Why, when, where and how? A national survey-based analysis. J Glob Health.

[36] Perkins JE, Rahman AE, Siddique AB, Haider MR, Banik G, Tahsina T (2019). Opting for home birth in rural Bangladesh: an assessment of the current status and reasons. Birth.

[37] Saha M, Odjidja EN (2017). Access to a skilled birth attendant in Bangladesh: what we know and what health system framework can teach us. Heal Syst Policy Res.

[38] Hasan F, Alam MM, Hossain MG (2019). Associated factors and their individual contributions to caesarean delivery among married women in Bangladesh: analysis of Bangladesh Demographic and Health Survey data. BMC Pregnancy Childbirth.

[39] Faruk MO, Sultana S, Al-Neyma M, Hossain S (2023). Socioeconomic, demographic, and nutritional factors associated with cesarean deliveries among childbearing women in Bangladesh. J Med Surg Public Heal.

[40] Abdulla F, Hossain MM, Rahman MM, Rahman MS, Rahman A (2023). Risk factors of caesarean deliveries in urban–rural areas of Bangladesh. Front Reprod Heal.

[41] Blum LS, Sharmin T, Ronsmans C (2006). Attending home vs. clinic-based deliveries: perspectives of skilled birth attendants in Matlab, Bangladesh. Reprod Health Matters.

[42] Say L, Raine R (2007). A systematic review of inequalities in the use of maternal health care in developing countries: examining the scale of the problem and the importance of context. Bull World Health Organ.

[43] Chowdhury MAH, Hasan MM, Ahmed S, Darwin C, Hasan MS, Haque MR (2013). Socio-demographic factors associated with home delivery assisted by untrained traditional birth attendant in rural Bangladesh. Am J Public Heal Res.

[44] Ryan BL, Krishnan RJ, Terry A, Thind A (2019). Do four or more antenatal care visits increase skilled birth attendant use and institutional delivery in Bangladesh? A propensity-score matched analysis. BMC Public Health.

[45] Kamal SM (2013). Preference for institutional delivery and caesarean sections in Bangladesh. J Heal Popul Nutr.

[46] Adanu RMK (2010). Utilization of obstetric services in Ghana between 1999 and 2003. Afr J Reprod Health.

[47] National Institute of Population Research and Training, Mitra and Associates, ICF International. Bangladesh Demographic and Health Survey 2014. Demogr. Heal. Surv. Dhaka, Bangladesh, and Rockville, Maryland, USA: NIPORT, Mitra and Associates, and ICF International; 2016.

[48] Spangler SA, Bloom SS (2010). Use of biomedical obstetric care in rural Tanzania: the role of social and material inequalities. Soc Sci Med.

[49] Stephenson R, Elfstrom KM (2012). Community influences on antenatal and delivery care in Bangladesh, Egypt, and Rwanda. Public Health Rep.

[50] De Allegri M, Ridde V, Louis VR, Sarker M, Tiendrebéogo J, Yé M (2011). Determinants of utilisation of maternal care services after the reduction of user fees: a case study from rural Burkina Faso. Health Policy.

[51] Collin SM, Anwar I, Ronsmans C (2007). A decade of inequality in maternity care: antenatal care, professional attendance at delivery, and caesarean section in Bangladesh (1991–2004). Int J Equity Health.

[52] Pervin J, Moran A, Rahman M, Razzaque A, Sibley L, Streatfield PK (2012). Association of antenatal care with facility delivery and perinatal survival—a population-based study in Bangladesh. BMC Pregnancy Childbirth.

[53] Ahinkorah BO, Seidu AA, Budu E, Agbaglo E, Appiah F, Adu C (2021). What influences home delivery among women who live in urban areas? Analysis of 2014 Ghana Demographic and Health Survey data. PLoS ONE.

[54] Devkota B, Maskey J, Pandey AR, Karki D, Godwin P, Gartoulla P (2020). Determinants of home delivery in Nepal—a disaggregated analysis of marginalised and non-marginalised women from the 2016 Nepal Demographic and Health Survey. PLoS ONE.

[55] Yaya S, Bishwajit G, Ekholuenetale M. Factors associated with the utilization of institutional delivery services in Bangladesh. PLoS One. 2017;12(2):e0171573. 10.1371/journal.pone.0171573 PMC5305190 28192478

[56] Kitui J, Lewis S, Davey G (2013). Factors influencing place of delivery for women in Kenya: an analysis of the Kenya demographic and health survey, 2008/2009. BMC Pregnancy Childbirth.

[57] Andersen RM (1995). Revisiting the behavioral model and access to medical care: Does it matter?. J Health Soc Behav.

[58] Dalal K, Shabnam J, Andrews-Chavez J, Mårtensson LB, Timpka T. Economic empowerment of women and utilization of maternal delivery care in Bangladesh. Int J Prev Med. 2012;3(9):628–36. PMC3445279 23024852

[59] Rahman M (2009). Deliveries among adolescent mothers in rural Bangladesh: Who provides assistance?. World Health Popul.

[60] Prata N, Bell S, Holston M, Quaiyum MA. Is attendant at delivery associated with the use of interventions to prevent postpartum hemorrhage at home births? The case of Bangladesh. BMC Pregnancy Childbirth. 2014;14:24. 10.1186/1471-2393-14-24 PMC3898406 24428902

[61] Pulok MH, Sabah MN, Uddin J, Enemark U. Progress in the utilization of antenatal and delivery care services in Bangladesh: where does the equity gap lie? BMC Pregnancy Childbirth. 2016;16(1):200. 10.1186/s12884-016-0970-4 PMC4967314 27473150

[62] Shahabuddin A, Nöstlinger C, Delvaux T, Sarker M, Delamou A, Bardají A (2017). Exploring maternal health care-seeking behavior of married adolescent girls in Bangladesh: a social-ecological approach. PLoS ONE.

[63] Goodburn EA, Gazi R, Chowdhury M (1995). Beliefs and practices regarding delivery and postpartum maternal morbidity in rural Bangladesh. Stud Fam Plan.

[64] Banke-Thomas OE, Banke-Thomas AO, Ameh CA (2017). Factors influencing utilisation of maternal health services by adolescent mothers in Low-and middle-income countries: a systematic review. BMC Pregnancy Childbirth.

[65] Kyei-Nimakoh M, Carolan-Olah M, McCann TV (2017). Access barriers to obstetric care at health facilities in sub-Saharan Africa-a systematic review. Syst Rev.

